# VxrB Influences Antagonism within Biofilms by Controlling Competition through Extracellular Matrix Production and Type 6 Secretion

**DOI:** 10.1128/mbio.01885-22

**Published:** 2022-07-26

**Authors:** Jennifer K. Teschler, Eva Jiménez-Siebert, Hannah Jeckel, Praveen K. Singh, Jin Hwan Park, Stefan Pukatzki, Carey D. Nadell, Knut Drescher, Fitnat H. Yildiz

**Affiliations:** a Department of Microbiology and Environmental Toxicology, University of California, Santa Cruz, Santa Cruz, USA; b Biozentrum, University of Basel, Basel, Switzerland; c Max Planck Institute for Terrestrial Microbiology, Marburg, Germany; d Department of Physics, Philipps University Marburg, Marburg, Germany; e Department of Biology, The City College of New York, New York, New York, USA; f Department of Biological Sciences, Dartmouth College, Hanover, New Hampshire, USA; University of Hawaii at Manoa

**Keywords:** *Vibrio cholerae*, biofilm, type six secretion system

## Abstract

The human pathogen Vibrio cholerae grows as biofilms, communities of cells encased in an extracellular matrix. When growing in biofilms, cells compete for resources and space. One common competitive mechanism among Gram-negative bacteria is the type six secretion system (T6SS), which can deliver toxic effector proteins into a diverse group of target cells, including other bacteria, phagocytic amoebas, and human macrophages. The response regulator VxrB positively regulates both biofilm matrix and T6SS gene expression. Here, we directly observe T6SS activity within biofilms, which results in improved competition with strains lacking the T6SS. VxrB significantly contributes to both attack and defense via T6SS, while also influencing competition via regulation of biofilm matrix production. We further determined that both *Vibrio* polysaccharide (VPS) and the biofilm matrix protein RbmA can protect cells from T6SS attack within mature biofilms. By varying the spatial mixing of predator and prey cells in biofilms, we show that a high degree of mixing favors T6SS predator strains and that the presence of extracellular DNA in V. cholerae biofilms is a signature of T6SS killing. VxrB therefore regulates both T6SS attack and matrix-based T6SS defense, to control antagonistic interactions and competition outcomes during mixed-strain biofilm formation.

## INTRODUCTION

Bacteria within biofilms engage in many physical and chemical interactions that can range from cooperation to antagonism (1, 2). These interactions drive the emergent properties of biofilms, including interstrain spatial architecture, resource competition and capture, and resistance to stressors (2). Common cooperative interactions in biofilms are metabolite cross-feeding and the production of the extracellular matrix. Depending on the biophysical properties of the matrix, which vary across strains and species, matrix secretion can also mediate spatial competition between different lineages that differentially produce it (1, 3). An extreme type of antagonistic interaction is caused by the type six secretion system (T6SS), which is found in the genomes of approximately 25% of sequenced Gram-negative bacteria. The T6SS requires cell-to-cell contact to deliver toxic effectors, resulting in the target cell’s death if it does not possess corresponding immunity factors (4), which is predicted to ultimately result in a competitive hierarchy that locally enriches for the attacking strain over susceptible strains (1, 5–7).

While cooperative interactions in biofilms have been investigated comprehensively, the impact of T6SS on biofilm formation is still being explored. For V. cholerae biofilms it has recently been shown that *Vibrio* polysaccharide (VPS)—a central component of the V. cholerae biofilm matrix—can protect cells against exogenous T6SS attack from other species without preventing V. cholerae from utilizing its own T6SS (8). Despite the VPS-based protection, T6SS killing can generate clonal patches of competing strains, even within initially well-mixed colonies (9). When V. cholerae mixed-strain biofilms are formed in confined spaces, killing via the T6SS eventually becomes limited due to the accumulation of dead cells along borders of strain groups (10). This consolidates spatial separation between the strains and allows T6SS-susceptible cells to coexist in biofilms along with a T6SS-active predator (10, 11). Additionally, the V. cholerae T6SS plays an important role in invading and displacing the microbiota to colonize the small intestine (12–15). Although regulation of the T6SS in V. cholerae has been studied in some detail (4), the role of T6SS regulators during biofilm formation is not well understood. Notably, the response regulator VxrB influences both the T6SS and biofilm matrix production, indicating that matrix production and T6SS activity may be intertwined in their effect on the ecology of antagonism during biofilm formation (12, 16).

The spatial distribution of T6SS activity within biofilms and the population dynamic consequences at cellular resolution are unknown. Similarly, though transcriptional profiling shows that T6SS genes are upregulated in biofilms (17), the interaction of T6SS activity and other mechanisms mediating competition in biofilms is not well characterized. Here, we characterize T6SS firing and killing within V. cholerae biofilms using high-resolution imaging, first focusing on the role of the response regulator VxrB and the role of particular biofilm components in altering competition dynamics. We then demonstrate a critical effect of the population structure and the degree of spatial mixing among strains for the efficacy of T6SS-mediated antagonism. Finally, we show that T6SS killing within biofilms creates an increased release of extracellular DNA (eDNA) in the vicinity of killed cells, which may have downstream consequences for biofilm architecture.

## RESULTS AND DISCUSSION

### Direct visualization of T6SS activity in biofilms.

**FIG 1:**
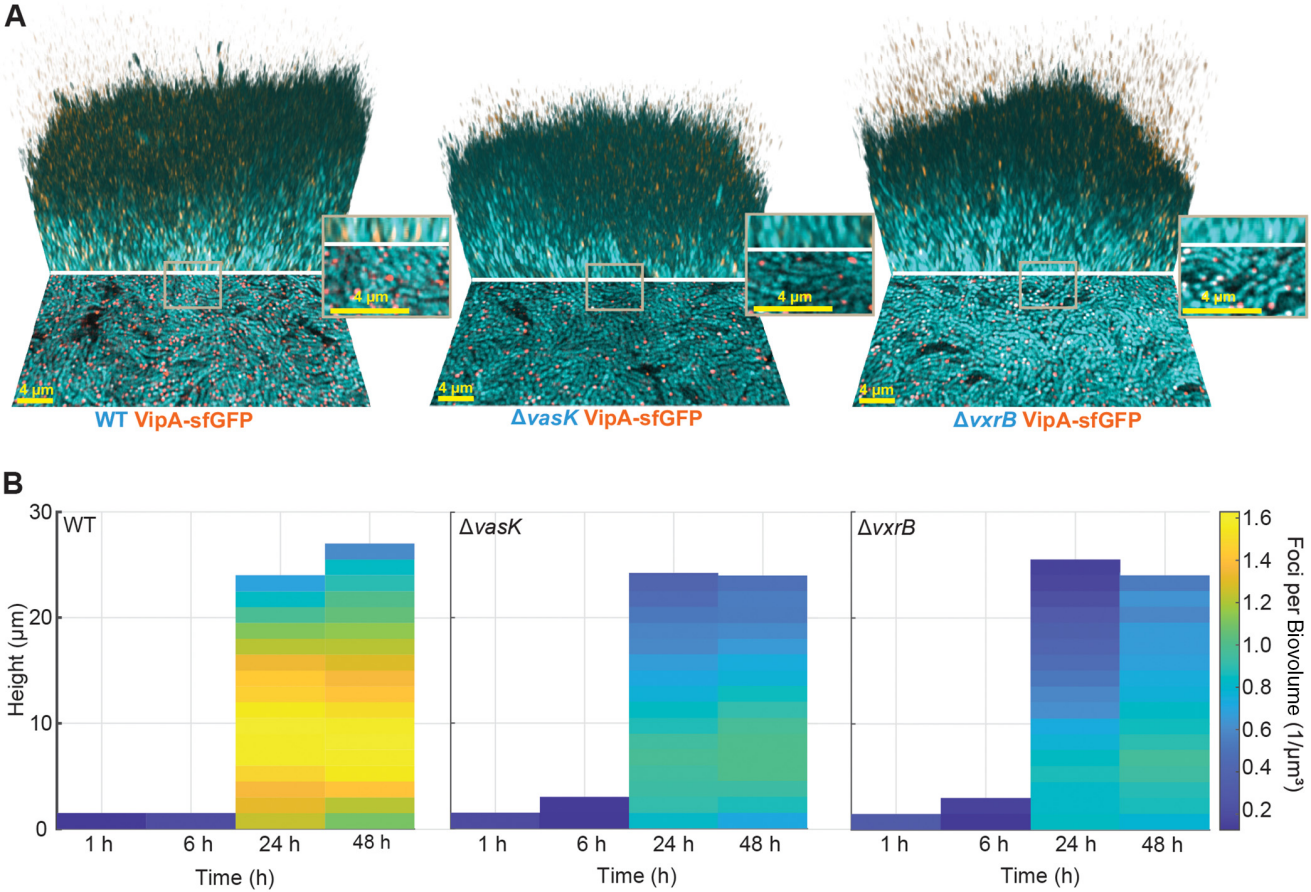
VxrB regulates T6SS assembly in mature biofilms. (A) Three-dimensional images of biofilms 48 h after inoculation. V. cholerae is depicted in cyan and VipA-sfGFP foci are shown in yellow, overlap of cyan and yellow is visualized as orange. The image for each strain shows the bottom layer of the biofilm (lower portion) and a three-dimensional rendering of the mature biofilm (upper portion). Scale bar is 4 μm. (B) Heatmaps depicting spatial distribution and quantity of VipA-sfGFP foci normalized to the biofilm biovolume at 1 h, 6 h, 24 h, and 48 h for WT, Δ*vasK*, and Δ*vxrB* strains. Heatmaps are the average of *n *= 6 biofilms, and the colorbar indicates the number of T6SS foci per biovolume (1/μm^3^).

To better understand when and where the T6SS is assembled within biofilms, we grew V. cholerae biofilms in flow chambers, using strains harboring a functional VipA-sfGFP fusion (18), and imaged the three-dimensional biofilms using confocal microscopy. VipA, along with VipB, forms the tubular structure of the outer sheath of the T6SS apparatus (19), and VipA-sfGFP can be used to visualize T6SS structures formed in living cells (18). We compared T6SS assembly during biofilm formation by V52 wild-type, V52 Δ*vasK*, and V52 Δ*vxrB* strains (Fig. 1A). VasK is an ATPase required for T6SS firing (its absence abolishes T6SS secretion, but not assembly [20–23]); VrxB has been shown to influence T6SS and biofilm matrix production (12, 16).

BiofilmQ was used to count VipA foci, allowing for quantitative spatial analysis of VipA production in each strain background (24). Wild type biofilms showed extensive expression and assembly of VipA-sfGFP foci by 24 h of growth, with the highest signal registered roughly 10 μm below the outer boundary of the biofilm with the surrounding growth medium (Fig. 1B). We observed that the dual regulator for T6SS and biofilm matrix, VxrB, positively contributes to T6SS assembly during biofilm formation: the number of T6SS foci per biovolume was at least 2-fold lower in a Δ*vxrB* strain when compared to a wild-type strain in biofilms at 24 h and 48 h after inoculation. We saw a similar decrease in the number of T6SS foci per biovolume for the Δ*vasK* strain, in which the T6SS should assemble but not fire, compared to a wild-type strain (Fig. 1B). This is consistent with previous observations reporting that the loss of VasK leads to decreased transcription of T6SS genes (8) and suggests that there may be a positive regulatory feedback mechanism between T6SS firing and reassembly.

### T6SS killing can be observed within biofilms.

**FIG 2:**
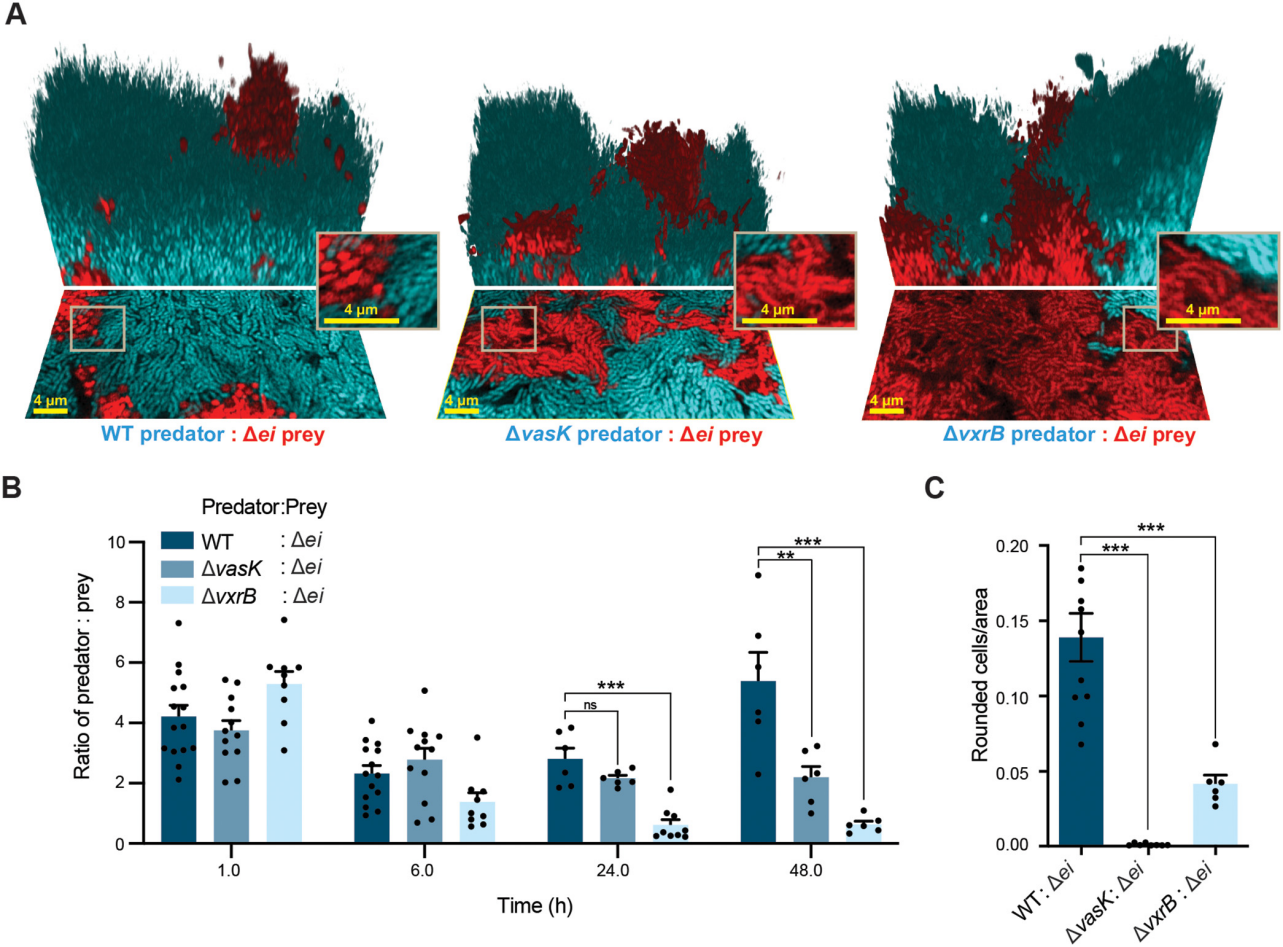
T6SS killing in mature biofilms. (A) Three-dimensional images of biofilms 48 h after inoculation. The V. cholerae predator strain is depicted in cyan, and the V. cholerae prey strain (Δ*ei* indicates that the T6SS effector immunity pairs have been deleted) is depicted in red. Image shows the bottom layer of the biofilm (lower portion) and a three-dimensional rendering of the mature biofilm (upper portion). Scale bar is 4 μm. (B) Predator to prey ratio in 1 h, 6 h, 24 h, and 48 h biofilms. (ns, no significance; **, *P < *0.005; ***, *P < *0.0001; *n *= 6). (C) Rounded prey cells normalized to total prey at the base of 48 h biofilms (***, *P < *0.0001; *n *= 6).

We next wanted to better understand how the T6SS impacted competition between strains during biofilm formation. To this end, we constructed a susceptible prey strain lacking the major T6SS effector/immunity protein pairs (referred to in figures as Δ*ei*, which is equivalent to Δ*tseLtsiV1*Δ*vasXtsiV2*Δ*vgrG3tsiV3*) (23, 25–27). The immunity genes are encoded directly downstream of their effector pair and code for proteins that can deactivate toxic effectors delivered by the T6SS, thus protecting the cell (25). The effector and immunity proteins are deleted together to prevent the endogenous effector production that would lead to self-lysis. The Δ*ei* strain is therefore susceptible to cell lysis by the T6SS attack from neighboring cells and has an inactive T6SS, as it also lacks the T6SS effector proteins. Pilot experiments indicated minimal T6SS-mediated killing of a Δ*ei* prey strain in the V. cholerae A1552 background by a wild-type (WT) V. cholerae A1552 predator strain. We therefore used V. cholerae V52 predator strains instead, as this strain background showed quantifiable killing of A1552 Δ*ei* prey cells. Control experiments in which WT A1552 and WT V52 were competed against each other in biofilms showed that the V52 strain background has a small intrinsic fitness disadvantage (Fig. S1 in the supplemental material), which may be due to differences in their investment into extracellular matrix (3, 28, 29). However, when grown in liquid media, no growth difference between predator and prey strains was observed (Fig. S6). Since VasK is required for T6SS killing, we used a Δ*vasK* deletion mutant as a T6SS-inactive control predator (20–23). We then used liquid cultures composed of a predator strain and the Δ*ei* prey strain mixed at a 5:1 ratio (predator:prey) at OD_600_ = 0.01 to inoculate flow chambers for subsequent mixed-strain biofilm growth. We imaged the resulting biofilms at different stages of biofilm formation: surface attachment (1h), microcolony formation (6 h), and mature biofilms (24h, 48h) (Fig. 2A, Movies S1–S3). From these images, the ratio of predator:prey was measured by computational image analysis. The predator:prey ratio was used as a metric of their competitive interaction over time, and the number of rounded cells normalized by the total number of prey cells was taken as a metric for T6SS-dependent killing. Rounded cells are a commonly used metric for detecting T6SS-killing, as cells have been shown to round in response to T6SS attack prior to lysis (30). The biofilm filling fraction, which is a measure of the cell density inside biofilms, does not differ between the predator and prey strains (Fig. S2A). The Δ*ei* prey cells primarily localize in the deeper regions of the biofilm and are overgrown by the predator cells (Figure S2B1, C1, D1). Most of the biomass of the biofilm is located in the deeper region of the biofilm, and the upper regions are less dense (Figure S2B2, C2, D2).

Noting that the WT V52 strain is at a slight disadvantage in biofilms (Fig. S1), the roughly constant predator:prey ratio for the WT predator in competition with Δ*ei* prey over 48 h (Fig. 2A and B) implies that the WT predator obtained a fitness benefit from the T6SS activity. We could also directly detect killing of prey cells via cell rounding in the presence of WT predator cells (Fig. 2C). By comparison, the Δ*vasK* control predator strain showed no measurable killing of prey (Fig. 2C), and the Δ*vasK* strain showed a marginal decrease in relative abundance in compeititon with the Δ*ei* prey strain, as expected from the small differences observed in control experiments with competitions between the WT V52 and WT A1552 strains (Fig. 2B). The Δ*vxrB* strain decreased substantially in relative abundance in competition with the Δ*ei* prey strain (Fig. 2B), and produced less cell death in the prey strain compared to the WT T6SS-capable strain (Fig. 2C). This result suggests that the response regulator VxrB contributes to the ability of V. cholerae to compete within mixed-strain biofilms and positively contributes to T6SS killing within biofilms.

### VxrB contributes to protection against T6SS killing in biofilms.

**FIG 3:**
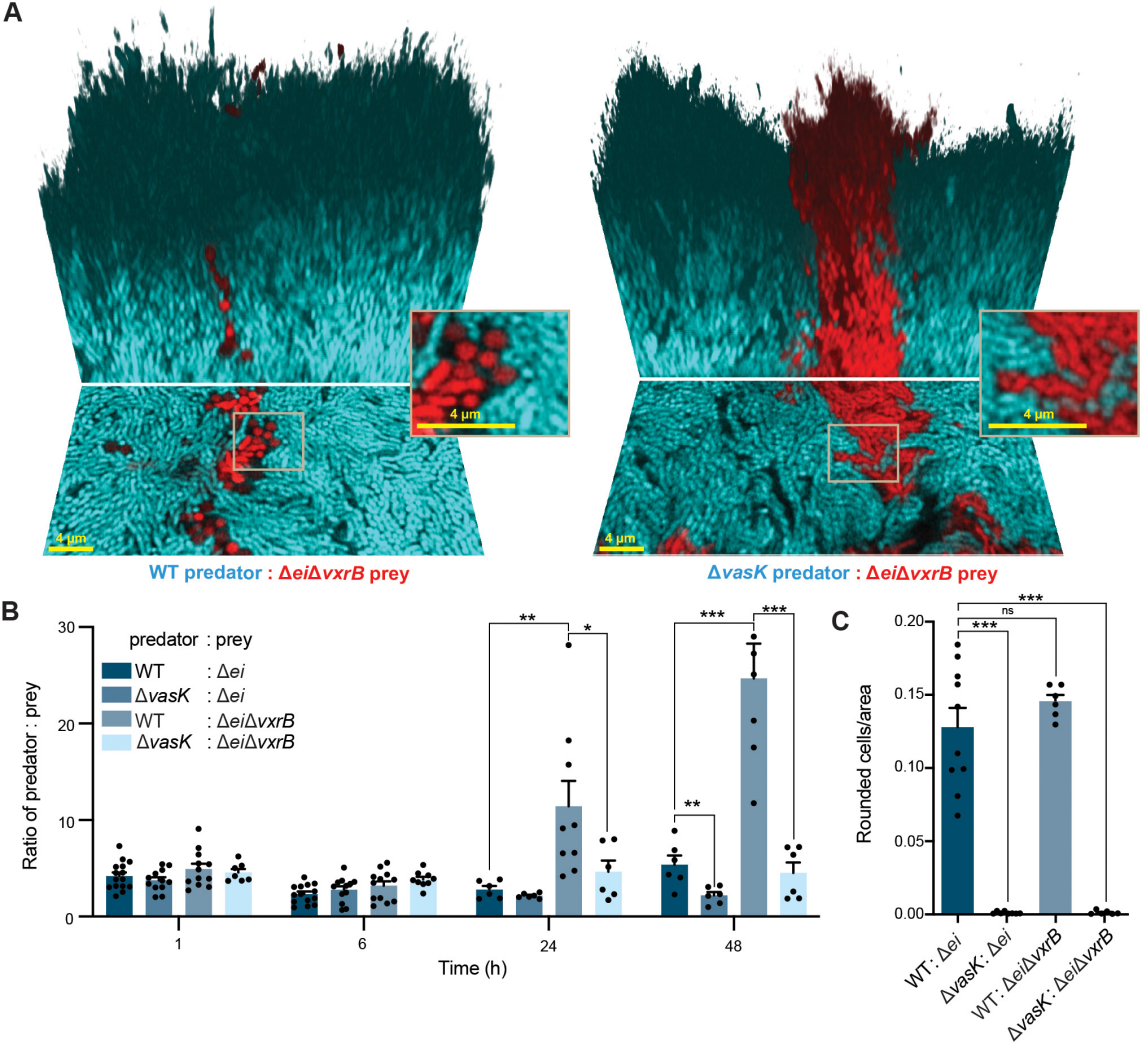
VxrB protects from T6SS killing in mature biofilms. (A) Three-dimensional images of biofilms 48 h after inoculation. The V. cholerae predator strain is depicted in cyan, and the V. cholerae prey strain (Δ*ei* indicates that the T6SS effector immunity pairs have been deleted) is depicted in red. Image shows the bottom layer of the biofilm (lower portion) and a three-dimensional rendering of the mature biofilm (upper portion). Scale bar is 4 μm. (B) Predator to prey ratio in 1 h, 6 h, 24 h, and 48 h biofilms. (*, *P < *0.05; **, *P < *0.005; ***, *P < *0.0001; *n *= 6). (C) Number of rounded prey cells normalized to total area occupied by prey cells at the base of 48 h biofilms (ns, no significance; ***, *P < *0.0001; *n *= 6).

VxrB has been implicated in both regulating T6SS attack as well as protection from T6SS killing (12, 31). To explore the effect of VxrB activity in the prey population, we used the same biofilm culture conditions described above, competing a T6SS+ wild-type predator and a T6SS– Δ*vasK* predator with a T6SS-susceptible Δ*ei* prey strain lacking *vxrB* (Fig. 3A). The biofilm filling fraction does not differ between the predator and prey strains (Fig. S3A). The Δ*ei*Δ*vxrB* prey cells primarily localize in the deeper regions of the biofilm and are overgrown by the predator cells in a more prominent way than the Δ*ei* cells in Fig. 2, indicating a protective function of VxrB in T6SS-mediated killing in biofilms (Fig. S3B, C).

We found that in competition with a T6SS+ wild-type strain, a Δ*ei*Δ*vxrB* prey strain fairs far worse than a Δ*ei* prey strain with VxrB intact (Fig. 2B, 3A, B). Specifically the T6SS+ predator increases from an initial ratio of 5:1 to a final ratio of ~20:1 (after 48 h) against a prey strain lacking VxrB. This result was dependent on the T6SS system being active in the predator, as a Δ*vasK* T6SS– strain competed neutrally, i.e., it did not appreciably change in ratio in competition with Δ*ei*Δ*vxrB* prey. Curiously, however, the amount of prey cell rounding in experiments with a T6SS+ predator was not different between the Δ*ei* strain possessing or lacking VxrB (Fig. 3C), suggesting that there were similar levels of prey killing whether or not the prey strain was producing VxrB. This could potentially be due to limitations in our methodology, which does not quantify cells that have already lysed and lost fluorescent signal, or due to the somewhat protective nature of dead cell debris along the interfaces between predator and prey strains (10). We suggest that the competitive defect of the Δ*ei*Δ*vxrB* prey strain can be primarily attributed to the loss of VxrB’s positive regulation of matrix production in the prey strain (16, 31).

### Biofilm matrix components play a protective role against T6SS killing in biofilms.

**FIG 4:**
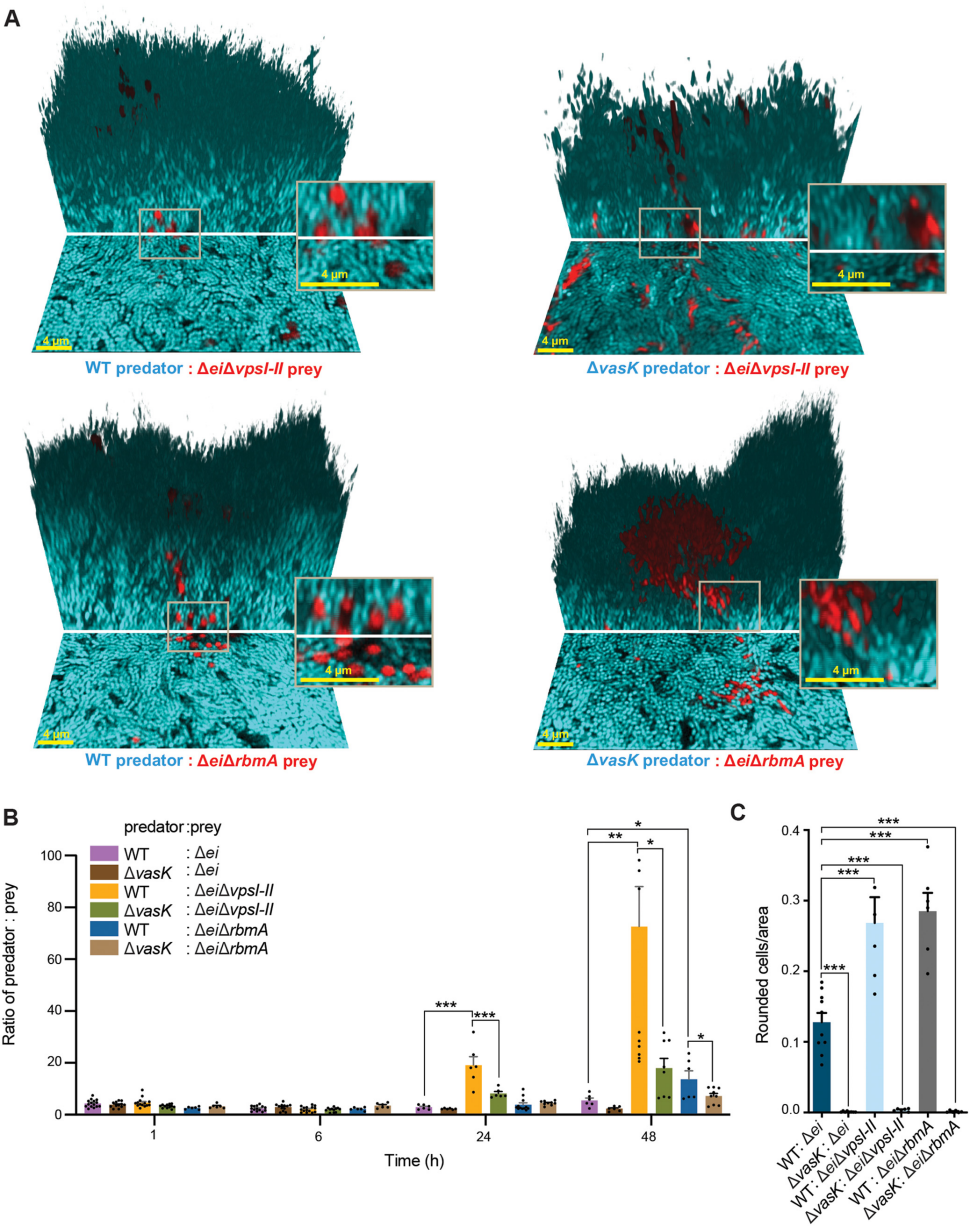
Biofilm components provide protection from T6SS killing in mature biofilms. (A) Three-dimensional images of biofilms 48 h after inoculation. The V. cholerae predator strain is depicted in cyan, and the V. cholerae prey strain (Δ*ei* indicates that the T6SS effector immunity pairs have been deleted) is depicted in red. Image shows the bottom layer of the biofilm (lower portion) and a three-dimensional rendering of the mature biofilm (upper portion). Scale bar is 4 μm. (B) Ratio of predator to prey in 1 h, 6 h, 24 h, and 48 h biofilms. (*, *P < *0.05; **, *P < *0.005; ***, *P < *0.0001; *n *= 6). (C) Number of rounded prey cells normalized to the total area occupied by prey cells at the base of 48 h biofilms (***, *P < *0.0001; *n *= 6).

Our competition data above suggest that VxrB activity within prey bacteria may influence their susceptibility to T6SS via regulation of biofilm matrix production; to test this possibility further, we compared the competition and T6SS killing activity against prey cells with and without key components of the V. cholerae biofilm matrix. The exopolysaccharide VPS and the secreted protein RbmA are important structural components of V. cholerae biofilms and contribute to cell–cell packing and formation of three-dimensional biofilm structures (32–34). Previous work identified a protective role for *Vibrio* polysaccharide (VPS) against T6SS attack, while suggesting that biofilm proteins did not lend the same protection (8). However, the capacity of these matrix components to modulate T6SS defense during mature biofilm formation between strains coinoculated within mixed biofilms is not yet clear. To examine this question, we deleted the two operons (*vps-*I and *vps-*II operons) encoding for *vps* genes in the Δ*ei* prey strain background. We also generated a *rbmA* deletion in the Δ*ei* prey strain background. We then competed these prey strains against a T6SS+ predator and a Δ*vasK* T6SS– control predator strain to isolate the effects of T6SS activity and differential matrix production on the susceptible prey (Fig. 4A). These comparisons were critical because differential VPS and RbmA production have been shown to contribute on their own to competitive success in mixed-strain biofilms of V. cholerae (3, 33, 35). The biofilm filling fraction does not differ between the predator and prey strains (Fig. S4A). The Δ*ei*Δ*vpsI-II* prey cells primarily localize in the deeper regions of the biofilm and are overgrown by the predator cells in a more prominent way than Δ*ei* cells, suggesting a protective role of VPS production in T6SS mediated killing in biofilms (Figure S4B1, C1, D1, E1). An increased intercell spacing resulting from the deletion of the *rbmA* gene in the Δ*ei* prey also leads to a decrease in competitive capacity of the Δ*ei*Δ*rbmA* prey, but it is not the predominant effect in matrix-mediated protection against the predator.

As noted in Fig. 2 and Fig. 3, a wild-type V52 T6SS+ predator does not appreciably increase in relative abundance over time against a Δ*ei* prey strain. By contrast, in competition with Δ*ei*Δ*vpsI-II* prey, T6SS+ V. cholerae increased from an initial ratio of 5:1 to a final ratio of over 60:1 over 48 h; a T6SS– (Δ*vasK*) predator strain, on the other hand, increased only to a final ratio of 30:1 (Fig. 4A and B). This latter result reflects the competitive defect of prey that fail to produce VPS, the core matrix polysaccharide, when in coculture with matrix-replete but T6SS– competitors. The additional increase in relative abundance of T6SS+ predators against Δ*ei*Δ*vpsI-II* prey, in turn, reflects the combined competitive defects of the prey’s failure to produce the core matrix polysaccharide VPS and the increased susceptibility to T6SS activity relative to a Δ*ei* prey background. This shows that in the absence of matrix production on the part of prey, its susceptibility to T6SS attack increased substantially. In other words, VPS contributes to competition both as a component of the biofilm matrix and as a defense from T6SS attack. This observation supports earlier work demonstrating that VPS is protective against T6SS killing (8) and, further, that VPS is protective within highly structured biofilms. T6SS killing of the Δ*ei*Δ*vpsI-II* prey strain, as measured by rounded cells, was also higher than that for a VPS-producing prey strain, further supporting that VPS can protect against T6SS killing within biofilms.

By contrast with VPS null mutants, which are unable to produce three-dimensional biofilm architecture, V. cholerae cells lacking the matrix protein RbmA are still able to produce biofilms, but they are structurally less robust and have characteristically reduced cell–cell packing relative to biofilms of wild-type strains (3, 32, 36–40). In competition with Δ*ei*Δ*rbmA* prey cells, T6SS+ V. cholerae increased from an initial ratio of 5:1 to a final ratio of over 14:1 over 48 h; a T6SS– (Δ*vasK*) control predator strain, on the other hand, increased only to a final ratio of 7:1 (Fig. 4A and B). Consistent with previous reports (3), our results suggest that Δ*rbmA* prey mutants have a mild competitive deficiency against wild type in the absence of T6SS activity, most likely due to their reduced structural strength, but that Δ*rbmA* mutants are substantially more susceptible to T6SS killing compared with a prey strain that is able to produce all matrix components. This interpretation was corroborated by the observation that T6SS-dependent killing as measured by prey cell rounding was significantly higher against a prey strain lacking RbmA than for a prey strain producing the full set of matrix protein components (Fig. 4C). Previous work demonstrated that loss of *rbmA* leads to increased invasibility of planktonic cells into biofilms and reduced protection from Bdellovibrio bacteriovorus, as looser cell–cell packing allows the predatory bacteria to infiltrate the biofilm (3, 37). We suggest that a similar mechanism may contribute to the enhanced T6SS killing of the Δ*rbmA* prey strain; as the biofilm matures, the more loosely packed Δ*rbmA* cells may allow for greater access to prey cells by predator cells, leading to a higher susceptibility to killing.

### Increased spatial strain mixing greatly increases the efficacy of T6SS attack.

**FIG 5:**
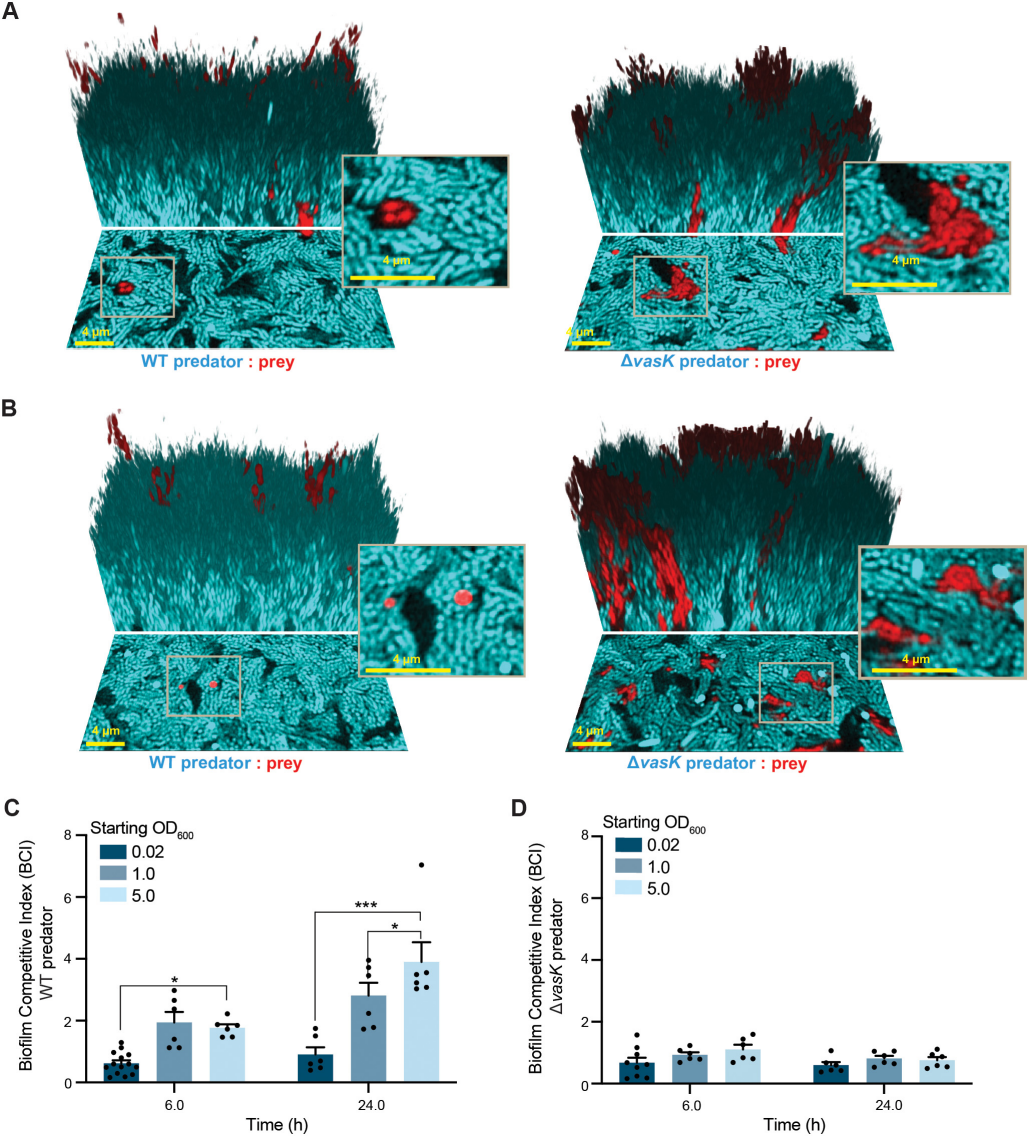
Increased contact during the early stages of biofilm formation favors a T6SS+ predator strain. (A) Three-dimensional images of 24-h biofilms formed with a high cell density of the starting inoculum (starting OD_600_ = 1.0), which results in increased early contact between predator and prey strains. The V. cholerae predator strain is depicted in cyan, and the V. cholerae prey strain (Δ*ei* indicates that the T6SS effector immunity pairs have been deleted) is depicted in red. The image shows the bottom layer of the biofilm (lower portion) and a three-dimensional rendering of the mature biofilm (upper portion). The scale bar is 4 μm. (B) Three-dimensional images of 24-h biofilms formed with a very high density of the starting inoculum (starting OD_600_ = 5.0) to enhance early contact between predator and prey strains. The V. cholerae predator strain is blue, and the V. cholerae prey strain is red. Image shows the bottom layer of the biofilm (lower portion) and a three-dimensional rendering of the mature biofilm (upper portion). (C) Biofilm competitive index (BCI) of WT predator strain competed against the prey strain, at 6 h, and at 24 h seeded with different inoculum densities (OD_600_ = 0.02, 1.0, 5.0; *n *= 6). BCI is defined as the final ratio of predator to prey bacteria at a particular time (6 h and 24 h) divided by the initial ratio of predator to prey bacteria. (*, *P < *0.05; ***, *P < *0.0001). (D) Biofilm competitive index (BCI) of Δ*vasK* predator strain competed against the prey strain at 6 h, and 24 h seeded with different inoculum densities (OD_600_ = 0.02, 1.0, 5.0; *n *= 6). BCI is defined as the final ratio of predator to prey bacteria at a particular time (6 h and 24 h) divided by the initial ratio of predator to prey bacteria.

Our results above demonstrate that strong effects of T6SS-mediated killing can be seen under conditions in which prey bacteria are not producing biofilm matrix components, making them much more susceptible to killing than matrix-producing prey, which were less affected by the T6SS predator. We were surprised by the modest population dynamical effects of T6SS predation against the prey, and so we next explored how varying the population spatial structure might alter this result. Under the biofilm conditions used for the previously described experiments, the cell density of the initial seeding inoculum was relatively low (OD_600_ ~0.02). These conditions allow for individual bacteria to attach to a surface and form spatially separated microcolonies before coming into contact with neighboring colonies composed of clonal lineages, reducing the total amount of contact area between T6SS+ and susceptible prey. Theory suggests that the degree of spatial mixing between strains, which controls the amount of contact area between predator cells and susceptible prey, should be a critical influence on the relative success of contact-mediated antagonism (1, 9, 41). To test this concept in our system, we altered the degree of mixing of T6SS+ predator or Δ*vasK* T6SS– control predators and Δ*ei* prey cells by increasing the inoculum density used to initiate biofilm growth (inoculum OD_600_ = 1 in Fig. 5A, OD_600_ = 5 in Fig. 5B). These treatments increase the initial surface coverage of flow chambers with random distributions of predator and prey cells, with the degree of mixing and contact among predators and prey increasing with increasing inoculum density. The biofilm filling fraction does not differ between the predator and prey strains at 24h, when the biofilms are grown from different initial surface seeding densities (Fig. S5).

Measuring the biofilm competitive index (defined as the final ratio of predator to prey bacteria at time x divided by the initial ratio of predator to prey bacteria) of T6SS+ predators to Δ*ei* prey over 24 h of biofilm growth, we found that the competitive advantage of T6SS+ predators over prey increased substantially with increasing initial inoculum density and initial biofilm surface coverage (Fig. 5C, initial attachment patterns of cells shown in Fig. S7). As noted above, with an initial inoculum OD_600_ of 0.02, T6SS+ predators do not appreciably increase in relative abundance over Δ*ei* prey over the course of 24 h. By contrast, with initial inocula of OD_600_ = 1.0, the predator:prey ratio increases approximately 3-fold by 24 h, and with initial inocula of OD_600_ = 5.0, the predator:prey ratio increases approximately 4-fold by 24 h. These results were entirely dependent on T6SS activity, as control experiments with a T6SS– control predator (harboring a Δ*vasK* deletion) showed minimal change in the biofilm competition index, regardless of initial inoculum density.

### T6SS killing results in altered eDNA localization and release in biofilms.

Beyond influencing competition, T6SS killing within biofilms is expected to result in additional consequences from the cell lysis of the prey strain that may contribute to biofilm structure, available nutrients, or cell signaling. One of the expected by-products of T6SS killing is the release of eDNA via cell lysis. eDNA is an important structural component of the V. cholerae biofilm matrix (42) and additionally represents a potential source of nutrients and genetic material for horizontal gene transfer (43, 44). Therefore, we next analyzed the concentration of eDNA in our mixed strain biofilms.

**FIG 6:**
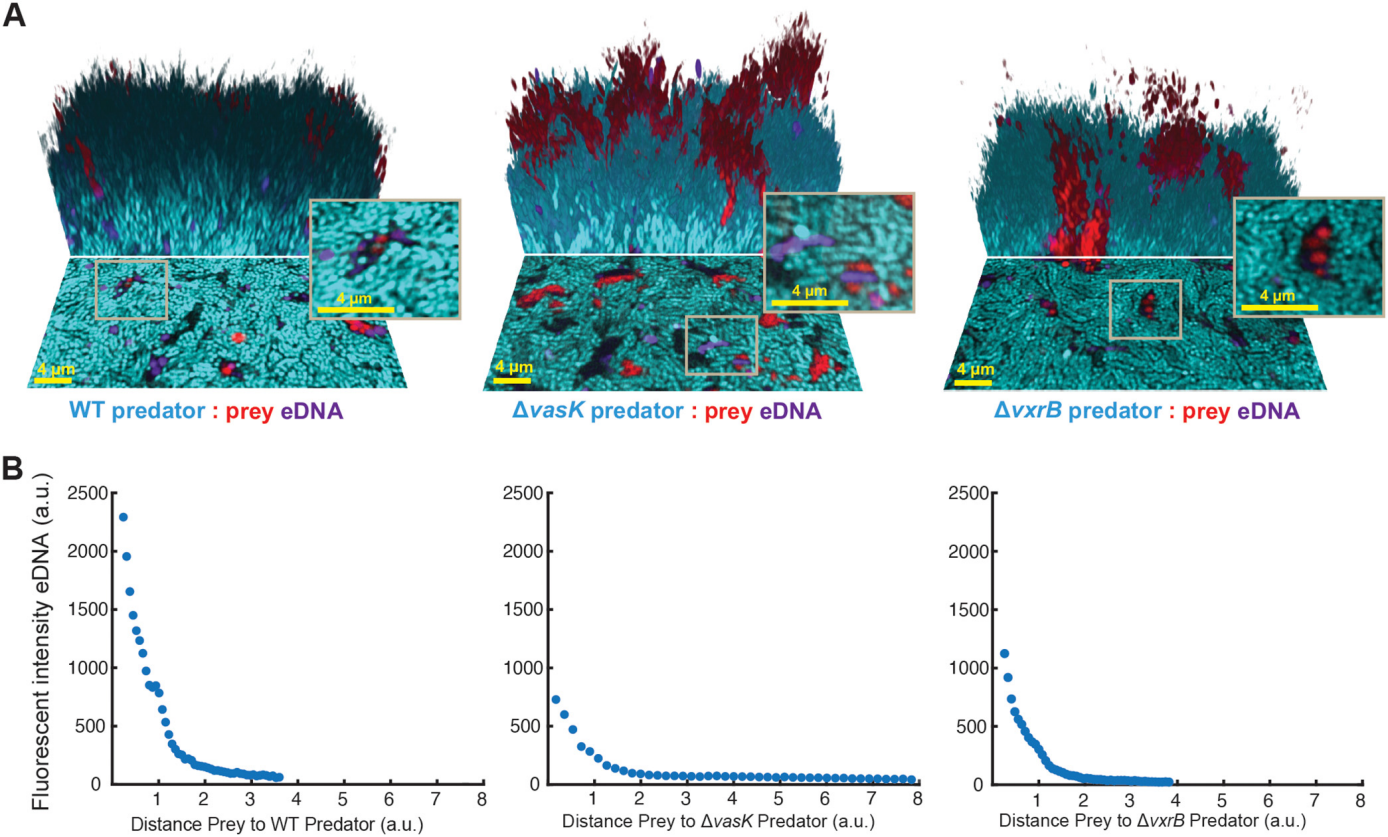
Extracellular DNA release is altered by T6SS killing. (A) Three-dimensional images of 24-h biofilms formed following seeding with a high cell density of the starting inoculum (starting OD_600_ = 1.0) to enhance early contact between predator and prey strains. The V. cholerae predator strain is depicted in cyan. The V. cholerae prey strain (Δ*ei* indicates that the T6SS effector immunity pairs have been deleted) is red, and extracellular DNA (eDNA, visualized using the TOTO-3 stain) is purple. The image shows the bottom layer of the biofilm (lower portion) and a three-dimensional rendering of the mature biofilm (upper portion). The scale bar is 4 μm. (B) Mean fluorescent intensity of eDNA measured as a function of the distance of the prey strain from the predator strain within biofilms after 24 h of growth (*n *= 12).

Using the TOTO-3 to stain for eDNA under the high-density starting inoculum conditions (OD_600_ = 1), we imaged biofilms after 24 h of growth. In biofilms formed with the T6SS+ predator, we observed a similar pattern of enhanced eDNA at the border between predator and prey strains, indicating that cell death by T6SS killing leads to DNA release in the biofilm (Fig. 6A). This border protects against continued killing within confined biofilms, as the accumulation of cell debris acts as a physical barrier between predator and prey strains (10). We observed that the eDNA appeared to maintain a spherical shape, similar to rounded cells in the process of dying. While this can be partially attributed to loss of membrane integrity in dying cells, this is consistent with reports of intact eDNA released in Pseudomonas aeruginosa biofilms that, compared to fragmented eDNA, maintains a rounded shape and has different properties than the fragmented eDNA (45). While some eDNA was observed at the border between predator and prey strains in biofilms formed with a Δ*vxrB* predator, it was less than what was observed for the WT predator. This is consistent with the reduction in T6SS killing observed in biofilms formed with a VxrB predator (Fig. 2 and 5). In biofilms formed with a Δ*vasK* predator, eDNA was distributed throughout the biofilm (Fig. 6A). We quantified the spatial distribution of the eDNA signal and found that, consistent with our visual observations, the eDNA signal was highest at the interface between predator and prey strains in biofilms formed with a T6SS+ predator and drastically decreased in the Δ*vxrB* predator and Δ*vasK* predator backgrounds (Fig. 6B). These findings demonstrate that the T6SS can contribute to localized enhanced eDNA release within mixed-strain biofilms.

The ability to lyse target cells in addition to killing them makes the T6SS a more effective weapon, as it helps clear the “corpse barriers” that act as a protective wall for the cells inside these barriers (11). Fast-acting lytic toxins have been shown to contribute to more effective lysis and enhanced T6SS killing in bacterial communities. Taxonomic analysis of lytic toxins across bacterial species showed that V. cholerae encodes for two “slow-lysing” lytic toxins (VasX and TseH) (11). It is possible that delivery of a higher dosage of these slow-lysing toxins may hasten and enhance cell lysis, perhaps accounting for the decrease in killing and eDNA signal observed in a biofilm formed by a Δ*vxrB* predator.

Localized cell death has been shown to alter the structures of biofilms (42, 46–48), and eDNA is an important structural component of biofilms (49). Killing via the T6SS has been shown to result in dead cell debris, which presumably includes eDNA, when a susceptible strain is present in V. cholerae biofilms (10). In other species, studies have found that lysed cells can be used as nutrients: Bacillus subtilis can “cannibalize” lysed cells, utilizing the debris for growth, and P. aeruginosa produces extracellular DNases to use eDNA from lysed cells as a nutrient source (50–52). It has been shown that V. cholerae can utilize eDNA as its sole phosphate source (42, 53), suggesting that it may use eDNA released via T6SS as a nutrient source, as observed for other species. T6SS-mediated eDNA release has previously been shown to facilitate horizontal gene transfer in V. cholerae grown on chitin, implying that strain interfaces where T6SS killing occurs have the potential to be evolutionary hotbeds (44, 54). eDNA has additionally been shown to interact with VPS in V. cholerae biofilms (55), suggesting that eDNA release via T6SS could also play a structural role within biofilms.

### Conclusion.

Here, we establish that the T6SS is actively fired and can kill susceptible competitors within biofilms. The response regulator VxrB enhances the ability of both predator and prey strains to compete in mixed-strain biofilms, likely via its coregulation of T6SS and biofilm matrix genes. The T6SS confers a competitive advantage during biofilm formation, allowing strains with an active T6SS to better maintain their foothold within the biofilm compared to strains lacking a T6SS, VxrB, or biofilm matrix components. This advantage is enhanced when competing strains have enhanced physical contact with one another during the early stages of biofilm formation. T6SS killing results in localized regions of cell death and eDNA release, although further work is needed to better understand the contribution of eDNA to biofilm structure and emergent properties. This work provides new insights into how bacteria compete within complex communities and enhances our understanding of the contributions of the VxrB regulator, biofilm matrix components, and spatiogenetic structure to T6SS-mediated competition within V. cholerae biofilms.

## MATERIALS AND METHODS

### Strains and growth conditions.

V. cholerae predator strains used are derivatives of the V52 O37 serogroup strain (56). V. cholerae prey strains used are derivatives of the O1 biovar El Tor strain A1552 in which the three main effector immunity pairs have been deleted (referred to in figures as Δ*ei*, which is equivalent to Δ*tseLtsiV1*Δ*vasXtsiV2*Δ*vgrG3tsiV3*) (57): FY_VC_13334 (V52 O37 *vipA*::sfGFP, Sm^R^), FY_VC_13977 (V52 O37 *vipA*::sfGFP Δ*vasK*, Sm^R^), FY_VC_13674 (V52 O37 *vipA*::sfGFP Δ*vxrB*, Sm^R^), FY_VC_13326 (V52 O37 serogroup strain, Sm^R^), FY_VC_13330 (V52 O37 Δ*vasK*, Sm^R^), FY_VC_16511 (V52 O37 Δ*vxrB*, Sm^R^), FY_VC_16095 (A1552 O1 El Tor, Δ*tseLtsiV1*Δ*vasXtsiV2*Δ*vgrG3tsiV3*, Rif^R^), FY_VC_16117 (A1552 O1 El Tor, Δ*vxrBΔtseLtsiV1*Δ*vasXtsiV2*Δ*vgrG3tsiV3*, Rif^R^), FY_VC_16287 (A1552 O1 El Tor, Δ*vpsI-II*Δ*tseLtsiV1*Δ*vasXtsiV2*Δ*vgrG3tsiV3*, Rif^R^), and FY_VC_16392 (A1552 O1 El Tor, Δ*rbmA*Δ*tseLtsiV1*Δ*vasXtsiV2*Δ*vgrG3tsiV3*, Rif^R^).

V. cholerae and Escherichia coli strains were grown aerobically in Luria-Bertani (LB) broth (1% tryptone, 0.5% yeast extract, 1% NaCl), pH 7.5, at 30°C and 37°C, respectively. LB agar contained granulated agar (Difco) at 1.5% (wt/vol). Antibiotics were used, when necessary, at the following concentrations: ampicillin (Ap), 100 μg/mL; streptomycin (Sm), 50 μg/mL; rifampicin (Rif), 100 μg/mL; gentamicin (Gm), 15 μg/mL.

### Strain and plasmid construction.

Plasmids were constructed using standard cloning methods or the Gibson Assembly recombinant DNA technique (New England Biolabs, Ipswich, MA). Gene deletions were carried out using allelic exchange of the native open reading frame (ORF) with the truncated ORF, as previously described (58). Plasmids harboring fluorescent proteins were mated into V. cholerae using an E. coli *strain* harboring the conjugation plasmid pRK2013.

### Biofilm assays and staining.

Flow cells were inoculated by diluting overnight-grown cultures of V. cholerae strains harboring either sfGFP or mRuby3 by 1:200 (OD_600_ of 0.02). For growing mixed-strain biofilms, cells were mixed at a 5:1 ratio of predator to prey. For biofilms with a starting inoculum of a high cell density, the OD_600_ was normalized to an OD_600_ of either 1.0 or 5.0, and cells were mixed at a 1:1 ratio of predator:prey to increase initial contact between the strains. Cells were then injected into an Ibidi m-Slide VI0.4 (Ibidi 80601; Ibidi LLC, Verona, WI). After inoculation, the bacteria were allowed to adhere to the substrate surface at room temperature for 1 h with no flow. Then, flow of 2% (vol/vol) LB (0.2 g/L tryptone, 0.1 g/L yeast extract, 1% NaCl) was initiated at a rate of 7.5 mL/h and continued for up to 48 h. For biofilms in which eDNA was stained, a starting OD_600_ of 1.0 was used, and strains were mixed at a 1:1 ratio before being injected into an Ibidi m-Slide. After biofilms were formed under flow conditions for 24 h, flow was halted, biofilms were gently washed with 1X phosphate-buffered saline (PBS) three times and then stained with TOTO-3 (2.0 μM final concentration) for 30 min. Biofilms were then gently washed with PBS three times before imaging. Confocal laser scanning microscopy (CLSM) images of the biofilms were captured with a Zeiss 880 Confocal microscope equipped with the Airyscan Fast function. To obtain data for image analysis, at least three Z-stacks were taken at independent locations within at least two biofilm replicates (total *n *= 6–12).

### Biofilm imaging.

Live biofilms were images at 1 h, 6 h, 24 h, and 48 h with a 63× oil immersion, numerical aperture 1.4 objective using a Zeiss 880 Confocal microscope with Airyscan Fast. A 488-nm laser was used to excite sfGFP, and a 561-nm laser was used to excite mRuby3. For experiments where eDNA was imaged, a 647-nm laser was used to excite TOTO-3. Images were collected in either the standard mode or the Airyscan Fast mode. Biofilm images were generated for presentation in the figures using the Imaris software (Bitplane). Briefly, images were cropped to show single cells at high magnification. Fluorescence channels were adjusted to highlight cells and features within the biofilm (settings are included in the supplemental material). The cropping and adjustments were used for representation only, and all data analyses were performed on the raw data as described below. To show the lower level of the biofilm, an ortho slicer was used to show a single *Z*-slice of the biofilm. For the three-dimensional rendering of the biofilm, the volume was demonstrated in the blend mode. The clipping tool was then used to combine the single *Z*-slice and three-dimensional representation into a single representative image.

### Image analyses.

Quantitative image analysis was performed using BiofilmQ, a software tool that has been developed for measuring spatially-resolved biofilm properties (available at drescherlab.org/data/BiofilmQ) (24). Details about the BiofilmQ program can be found in the cited publication and website; below, a brief description of the individual analyses performed are included.

For the spatiotemporal quantification of sfGFP-tagged VipA in V. cholerae, a threshold was applied to the mRuby3 channel, which was manually selected for each image to adjust for different imaging conditions. To analyze and quantify the spatial distribution of the biovolume, the segmented biovolume in the mRuby3-channel was then sub-divided into cubes (side-length 0.5 μm) using BiofilmQ (24). To identify the individual foci, the edge detection algorithm based on seeded watershed segmentation, as described in (59) with modifications from (60), was applied to the sfGFP channel. Based on this segmentation of the sfGFP-channel, properties of VipA foci were quantified using BiofilmQ (total fluorescence intensity and size, for each VipA focus). The spatial distribution of foci in the biofilm was determined by counting the number of VipA foci per biovolume cube and normalizing with the corresponding biovolume in the cube. A VipA focus was assigned to be localized within a particular cube if the focus’ centroid was within the cube volume.

Additionally, the spatial distributions of foci properties were measured by averaging the properties of the foci in each cube. Quantifications from cubes with a similar height above the substrate were averaged to result in one pixel in the heatmaps shown in Fig. 1. Heatmaps of spatiotemporal foci quantifications were obtained by averaging over the biological and technical replicates (*n *= 6). In these heatmaps, spatiotemporal pixels with less than 3 replicates were eliminated from the graph.

For the measurements of the predator to prey ratios, the following steps were performed. The 488-nm and 561-nm channels were segmented, to determine the overall biovolume of each strain within the biovolume. The biovolume of the predator was then divided by the biovolume of the prey strain to determine the ratio of predator to prey for each biofilm that was acquired (*n *= 6). Statistical analysis of this data was performed using ANOVA and Dunnett’s multiple comparison test.

For the quantification and localization of eDNA, the following steps were performed. Super-resolution images were cropped into quarters to reduce the data size for efficient computational processing. The 488-nm and 561-nm channels were segmented, and the nearest neighbor parameter was run to determine the distance of prey from a predator. The fluorescent intensity parameter was run for the 647-nm channel to assess the intensity of eDNA signal. The BiofilmQ visualization tool was then used to create a “1.5D histogram,” plotting the distance of prey from predator along the *x* axis and the fluorescent intensity of the eDNA signal along the y axis. A total of *n *= 12 biofilm images were quartered, analyzed, and plotted.

10.1128/mbio.01885-22.1 FIG S1 WT A1552 outcompetes WT V52 strains during biofilm formation. (A) Three-dimensional images of biofilms 48 h after inoculation. The V. cholerae WT V52 strain is depicted in blue, and the V. cholerae WT A1552 strain is depicted in red. Image shows the bottom layer of the biofilm (lower portion) and a three-dimensional rendering of the mature biofilm (upper portion). Scale bar is 4 μm. (B) V. cholerae WT V52 to WT A1552 ratio in biofilms grown for 1 h, 6 h, 24 h, and 48 h. Download FIG S1, PDF file, 0.2 MB. Copyright © 2022 Teschler et al. 2022 Teschler et al. https://creativecommons.org/licenses/by/4.0/ This content is distributed under the terms of the Creative Commons Attribution 4.0 International license.

10.1128/mbio.01885-22.2 FIG S2 Quantifications of biofilm structure and strain composition for experiments from Fig. 2. (A) Biofilm filling fractions of regions occupied by predator cells and regions occupied by prey cells from the experiments shown in Fig. 2, calculated for each strain separately. The biofilm filling fraction for each strain is the detected biovolume divided by the volume enclosed by the hull of the biovolume of each strain. No substantial differences in filling fractions between the strains and time points are apparent. At the 1h time point, there were primarily individual cells, and no multicellular structures, so that the filling fraction is not well defined and therefore these data are not shown. (B) For the biofilms resulting from the competition of WT versus Δ*ei* strains, heatmaps show the spatial (*y* axis, height inside the biofilm) and temporal (*x* axis) change of parameters. Top (B1): the fraction of the biofilm biovolume occupied by the WT strain is shown in color. Bottom (B2): total fraction of the image occupied by the biofilm (WT and Δ*ei* cells together) is shown in color, as a function of height in the biofilm. The heatmaps show that there is less biomass in higher regions of the biofilm, and that the WT fraction of the biomass can also vary with height in the biofilm. The prey cells are predominantly localized in the deeper regions of the biofilm. (C) For the biofilms resulting from the competition of Δ*vasK* versus Δ*ei* strains, heatmaps show the spatiotemporal change in parameters, analogous to panel B. Top (C1): the fraction of the biofilm biovolume occupied by the Δ*vasK* strain is shown in color. Bottom (C2): total fraction of the image occupied by the biofilm (Δ*vasK* and Δ*ei* cells together) is shown in color. (D) For the biofilms resulting from the competition of Δ*vxrB* versus Δ*ei* strains, heatmaps show the spatiotemporal change of parameters. Top (D1): the fraction of the biofilm biovolume occupied by the Δ*vxrB* strain is shown in color. Bottom (D2): Total fraction of the image occupied by the biofilm (Δ*vxrB* and Δ*ei* cells together) is shown in color. Download FIG S2, PDF file, 0.3 MB. Copyright © 2022 Teschler et al. 2022 Teschler et al. https://creativecommons.org/licenses/by/4.0/ This content is distributed under the terms of the Creative Commons Attribution 4.0 International license.

10.1128/mbio.01885-22.3 FIG S3 Quantifications of biofilm structure and strain composition for experiments from Fig. 3. (A) Biofilm filling fractions of regions occupied by predator cells and regions occupied by prey cells from the experiments shown in Fig. 3, calculated for each strain separately. The biofilm filling fraction for each strain is the detected biovolume divided by the volume enclosed by the hull of the biovolume of each strain. No significant differences in filling fractions between the strains and time points are apparent. At the 1 h time point, there were primarily individual cells, and no multicellular structures, so that the filling fraction is not well defined, and therefore these data are not shown. (B) For the biofilms resulting from the competition of WT versus Δ*ei*Δ*vxrB* strains, heatmaps show the spatial (*y* axis, height inside the biofilm) and temporal (*x* axis) change of parameters. Top (B1): the fraction of the biofilm biovolume occupied by the WT strain is shown in color. Bottom (B2): total fraction of the image occupied by the biofilm (WT and Δ*ei*Δ*vxrB* cells together) is shown in color, as a function of height in the biofilm. The heatmaps show that there is less biomass in higher regions of the biofilm, and that the WT fraction of the biomass can also vary with height in the biofilm. The prey cells are predominantly localized in the deeper regions of the biofilm, even more so than in the WT versus Δ*ei* condition (see Fig. S2). (C) For the biofilms resulting from the competition of Δ*vasK* versus Δ*ei*Δ*vxrB* strains, heatmaps show the spatiotemporal change in parameters, analogous to panel B. Top: the fraction of the biofilm biovolume occupied by the Δ*vasK* strain is shown in color. Bottom: Total fraction of the image occupied by the biofilm (Δ*vasK* and Δ*ei*Δ*vxrB* cells together) is shown in color. Download FIG S3, PDF file, 0.3 MB. Copyright © 2022 Teschler et al. 2022 Teschler et al. https://creativecommons.org/licenses/by/4.0/ This content is distributed under the terms of the Creative Commons Attribution 4.0 International license.

10.1128/mbio.01885-22.4 FIG S4 Quantifications of biofilm structure and strain composition for experiments from Fig. 4. (A) Biofilm filling fractions of regions occupied by predator cells and regions occupied by prey cells from the experiments shown in Fig. 4, calculated for each strain separately. The biofilm filling fraction for each strain is the detected biovolume divided by the volume enclosed by the hull of the biovolume of each strain. No significant differences in filling fractions between the strains and time points are apparent. At the 1 h time point, there were primarily individual cells, and no multicellular structures, so that the filling fraction is not well defined, and therefore these data are not shown. The expected lower filling fraction for the Δ*ei*Δ*rbmA* mutant prey is not reflected in the graph as a result of the invasion of the prey-intercell space by the predator cells. (B) For the biofilms resulting from the competition of WT versus Δ*ei*Δ*vpsI-II* strains, heatmaps show the spatial (*y* axis, height inside the biofilm) and temporal (*x* axis) change of parameters. Top (B1): the fraction of the biofilm biovolume occupied by the WT strain is shown in color. Bottom (B2): total fraction of the image occupied by the biofilm (WT and Δ*ei*Δ*vpsI-II* cells together) is shown in color, as a function of height in the biofilm. The heatmaps show that there is less biomass in higher regions of the biofilm, and that the WT fraction of the biomass can also vary with height in the biofilm. The prey cells are predominantly localized in the deeper regions of the biofilm. The Δ*ei*Δ*vpsI-II* cells are outcompeted by the WT in a stronger manner than the Δ*ei* cells in the WT versus Δ*ei* condition (see Fig. S2), indicating that VPS production plays an important role in the protection against T6SS-mediated killing in biofilms. (C) For the biofilms resulting from the competition of Δ*vasK* versus Δ*ei*Δ*vpsI-II* strains, heatmaps show the spatiotemporal change in parameters, analogous to panel B. Top (C1): the fraction of the biofilm biovolume occupied by the Δ*vasK* strain is shown in color. Bottom (C2): total fraction of the image occupied by the biofilm (Δ*vasK* and Δ*ei*Δ*vpsI-II* cells together) is shown in color. (D) For the biofilms resulting from the competition of WT versus Δ*ei*Δ*rbmA* strains, heatmaps show the spatiotemporal change in parameters, analogous to panel B. Top (D1): the fraction of the biofilm biovolume occupied by the WT strain is shown in color. Bottom (D2): total fraction of the image occupied by the biofilm (WT and Δ*ei*Δ*rbmA* cells together) is shown in color. The Δ*ei*Δ*rbmA* cells are outcompeted by the WT in a stronger manner than the Δ*ei* cells in the WT versus Δ*ei* condition (see supplemental figure “Fig. S2”), but not as strongly as in the WT versus Δ*ei*Δ*vpsI-II* condition (panel B of the present figure). These results suggest that increased intercell spacing due the lack of the matrix protein RbmA enhances T6SS-mediated killing in biofilms, but high cell packing is not the predominant protective mechanism as compared to VPS production. (E) For the biofilms resulting from the competition of Δ*vasK* versus Δ*ei*Δ*rbmA* strains, heatmaps show the spatiotemporal change in parameters, analogous to panel B. Top: the fraction of the biofilm biovolume occupied by the Δ*vasK* strain is shown in color. Bottom: total fraction of the image occupied by the biofilm (Δ*vasK* and Δ*ei*Δ*rbmA* cells together) is shown in color. Download FIG S4, PDF file, 0.3 MB. Copyright © 2022 Teschler et al. 2022 Teschler et al. https://creativecommons.org/licenses/by/4.0/ This content is distributed under the terms of the Creative Commons Attribution 4.0 International license.

10.1128/mbio.01885-22.5 FIG S5 Quantifications of biofilm structure and strain composition for experiments from Fig. 5. (A) Biofilm filling fractions of regions occupied by predator cells and regions occupied by prey cells from the experiments shown in Fig. 5 in the WT predator condition, calculated for each strain separately. The biofilm filling fraction for each strain is the detected biovolume divided by the volume enclosed by the hull of the biovolume of each strain. No significant differences in filling fractions between the different conditions (differing in initial seeding density of the cells in the microfluidic channel) and time points are apparent at 24 h. Low filling fraction at 6 h for OD 1 and OD 5 results from unclear cell cluster identification due to the high seeding density. At the 1 h time point, there were primarily individual cells, and no multicellular structures, so that the filling fraction is not well defined, and therefore these data are not shown. (B) Biofilm filling fractions of regions occupied by predator cells and regions occupied by prey cells from the experiments shown in Fig. 5 in the Δ*vasK* predator condition, calculated for each strain separately. The biofilm filling fraction for each strain is the detected biovolume divided by the volume enclosed by the hull of the biovolume of each strain. No significant differences in filling fractions between the different conditions (differing in initial seeding density of the cells in the microfluidic channel) and time points are apparent at 24 h. Low filling fraction at 6 h for OD 1 and OD 5 results from unclear cell cluster identification due to the high seeding density. At the 1 h time point, there were primarily individual cells, and no multicellular structures, so that the filling fraction is not well defined, and therefore these data are not shown. Download FIG S5, PDF file, 0.2 MB. Copyright © 2022 Teschler et al. 2022 Teschler et al. https://creativecommons.org/licenses/by/4.0/ This content is distributed under the terms of the Creative Commons Attribution 4.0 International license.

10.1128/mbio.01885-22.6 FIG S6 Growth curve of predator and prey strains. Growth curves performed in a 96-well plate over 16 h in LB media demonstrate that there is no difference in growth rate or final culture density between predator and prey strains. Download FIG S6, PDF file, 0.3 MB. Copyright © 2022 Teschler et al. 2022 Teschler et al. https://creativecommons.org/licenses/by/4.0/ This content is distributed under the terms of the Creative Commons Attribution 4.0 International license.

10.1128/mbio.01885-22.7 FIG S7 Predator and prey strain initial attachment after 1 h incubation using different inoculation ODs. (A) Predator (blue) and prey (red) attachment to surface after 1 h when inoculating with an initial inoculum OD_600_ = 0.02. (B) Predator (blue) and prey (red) attachment to surface after 1 h when inoculating with an initial inoculum OD_600_ = 1. (C) Predator (blue) and prey (red) attachment to surface after 1 h when inoculating with an initial inoculum OD_600_ = 5. Download FIG S7, PDF file, 0.3 MB. Copyright © 2022 Teschler et al. 2022 Teschler et al. https://creativecommons.org/licenses/by/4.0/ This content is distributed under the terms of the Creative Commons Attribution 4.0 International license.

10.1128/mbio.01885-22.8 MOVIE S1 Three-dimensional view of 48 h biofilm, showing Z-slices moving from the base to the top of the biofilm. V52 wild-type predator shown in blue and A1552 Δ*ei* prey shown in red. Strains were initially inoculated at a 5:1 ratio. Download Movie S1, AVI file, 5.2 MB. Copyright © 2022 Teschler et al. 2022 Teschler et al. https://creativecommons.org/licenses/by/4.0/ This content is distributed under the terms of the Creative Commons Attribution 4.0 International license.

10.1128/mbio.01885-22.9 MOVIE S2 Three-dimensional view of 48 h biofilm, showing Z-slices moving from the base to the top of the biofilm. V52 Δ*vasK* predator shown in blue and A1552 Δ*ei* prey shown in red. Strains were initially inoculated at a 5:1 ratio. Download Movie S2, AVI file, 4.8 MB. Copyright © 2022 Teschler et al. 2022 Teschler et al. https://creativecommons.org/licenses/by/4.0/ This content is distributed under the terms of the Creative Commons Attribution 4.0 International license.

10.1128/mbio.01885-22.10 MOVIE S3 Three-dimensional view of 4 8h biofilm, showing Z-slices moving from the base to the top of the biofilm. V52 Δ*vxrB* predator shown in blue and A1552 Δ*ei* prey shown in red. Strains were initially inoculated at a 5:1 ratio. Download Movie S3, AVI file, 3.9 MB. Copyright © 2022 Teschler et al. 2022 Teschler et al. https://creativecommons.org/licenses/by/4.0/ This content is distributed under the terms of the Creative Commons Attribution 4.0 International license.

Imaris software (Bitplane) was used to manually count rounded cells at the base of biofilms. This was done as follows. First, 3D biofilm images were cropped to include only a single Z-plane at the base of the biofilm. The spots tool was used to manually label and count all circular cells, and the surface tool was used to calculate the total area of the prey strain. To determine the ratio of round cells to total prey present, the number of round cells was divided by the total area of prey strain present (*n *= 6). Statistical analysis of this data was performed using ANOVA and Dunnett’s multiple comparison test.

For the quantification of the biofilm filling fraction, a foreground–background biovolume segmentation was performed on each fluorescence channel using Otsu’s method for threshold determination with a manually adjusted sensitivity. To compute the filling fraction, the detected biovolume was divided by the volume enclosed by the biofilm perimeter, which was calculated for each fluorescence channel individually. To determine the biofilm perimeter, three morphological image processing operations were performed on every slice of the super-resolution images: dilation of the detected biovolume using a disk structuring element with a 30-pixel radius (1.3 μm), followed by a hole-filling operation and three erosion steps using a disk structuring element with a radius of 10 pixels. The hull volume was corrected by subtracting the volume occupied by the other strain and reinclusion of the volume of the analyzed strain.

The spatiotemporal characterization of the predator biovolume fraction was based on the segmented data for the biofilm filling fraction quantification and was calculated by dividing, for each image slice of the 3D image stack, the area occupied by the predator by the total area detected for all bacterial cells. For the total biovolume image fraction quantification, for each slice the area occupied by bacterial cells was divided by the entire image area. For the analysis of biofilm-internal structures based on super-resolution images, 6 replicates are available for each condition, except for the Δ*vasK* versus Δ*ei* cocultures, where only 2 replicate super-resolution images are available. For computing the heatmaps, averages for each heatmap pixel were calculated for all biological replicates.
