# Supplementary material for: VxrB Influences Antagonism within Biofilms by Controlling Competition through Extracellular Matrix Production and Type 6 Secretion
Source: mBio. 2022 Jul 26;13(4):e01885-22. doi: 10.1128/mbio.01885-22 (PMC9426512; doi:10.1128/mbio.01885-22)
Supplement: FIG S1 [file mbio.01885-22-s0001.pdf]

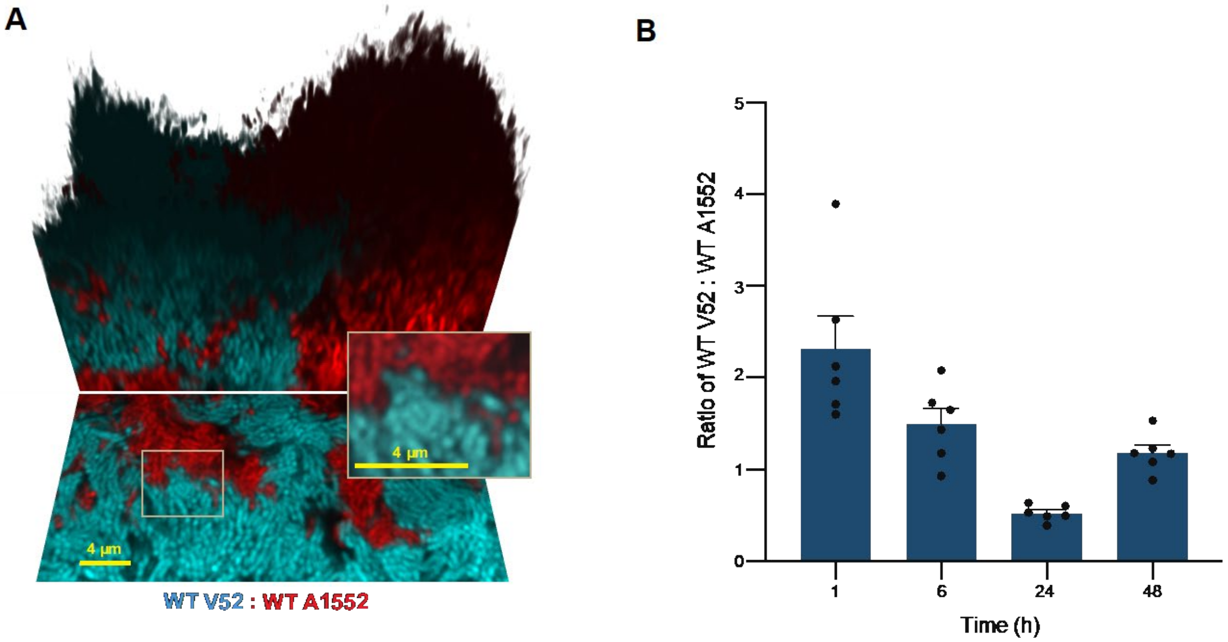

Figure S1. WT A1552 outcompetes WT V52 strains during biofilm formation. A) Three-dimensional images of biofilms 48 h after inoculation. The *V. cholerae* WT V52 strain is depicted in blue, and the *V. cholerae* WT A1552 strain is depicted in red. Image shows the bottom layer of the biofilm (lower portion) and a three-dimensional rendering of the mature biofilm (upper portion). Scale bar is 4 µm. B) *V. cholerae* WT V52 to WT A1552 ratio in biofilms grown for 1 h, 6 h, 24 h, and 48 h.
