# Supplementary material for: VxrB Influences Antagonism within Biofilms by Controlling Competition through Extracellular Matrix Production and Type 6 Secretion
Source: mBio. 2022 Jul 26;13(4):e01885-22. doi: 10.1128/mbio.01885-22 (PMC9426512; doi:10.1128/mbio.01885-22)
Supplement: FIG S2 [file mbio.01885-22-s0002.pdf]

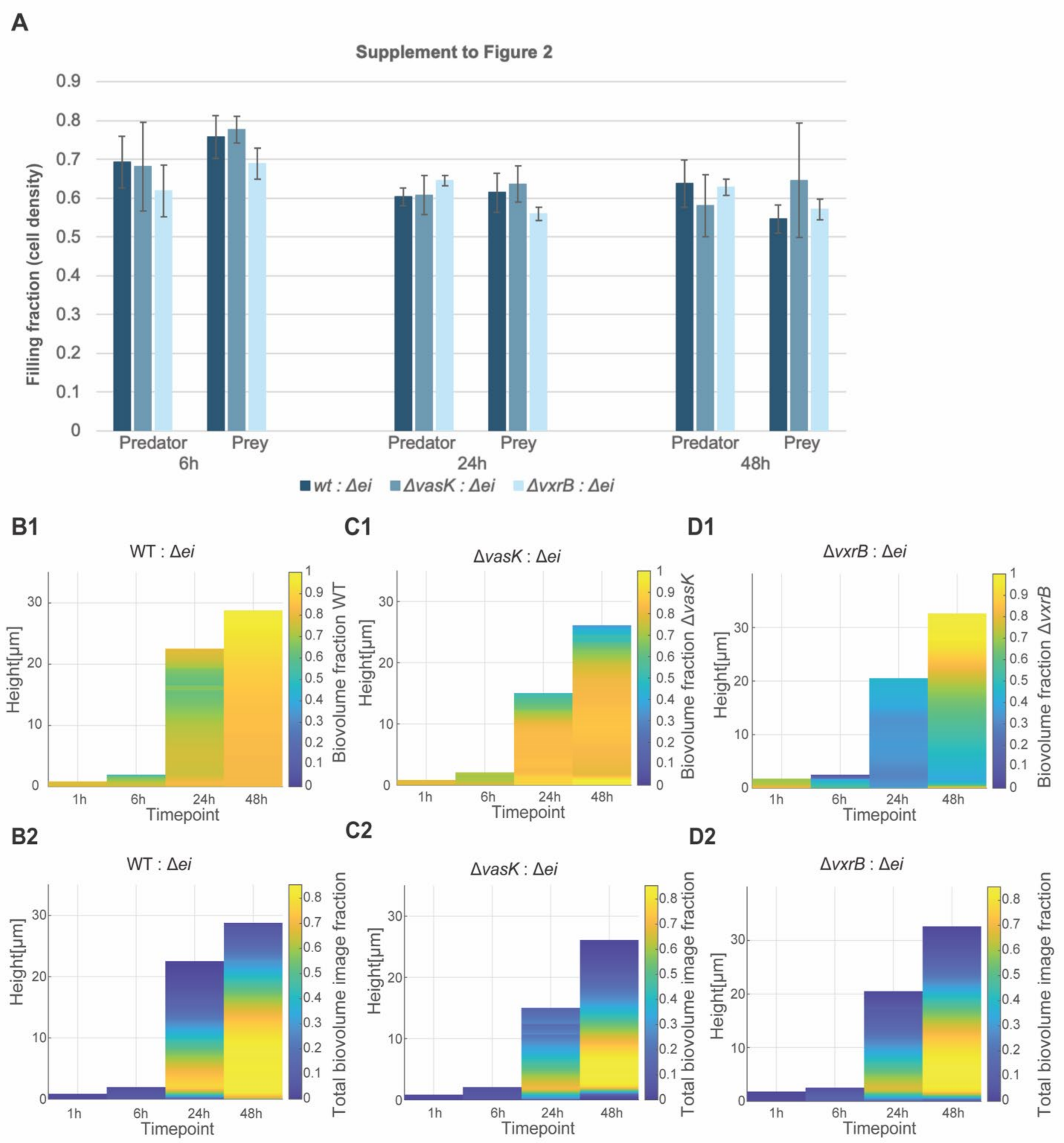

Figure S2. Quantifications of biofilm structure and strain composition for experiments from Figure 2. A) Biofilm filling fractions of regions occupied by predator cells and regions occupied by prey cells from the experiments shown in Figure 2, calculated for each strain separately. The biofilm filling fraction for each strain is the detected biovolume divided by the volume enclosed by the hull of the biovolume of each strain. No substantial differences in filling fractions between the strains and time points are apparent. At the 1h time point, there were primarily individual cells, and no multicellular structures, so that the filling fraction is not well defined and therefore these data are not shown. B) For the biofilms resulting from the competition of WT vs. Δ*ei* strains, heatmaps show the spatial (y-axis, height inside the biofilm) and temporal (x-axis) change of parameters. Top (B1): the fraction of the biofilm biovolume occupied by the WT strain is shown in color. Bottom (B2): Total fraction of the image occupied by the biofilm (WT and Δ*ei* cells together), is shown in color, as a function of height in the

biofilm. The heatmaps show that there is less biomass in higher regions of the biofilm, and that the WT fraction of the biomass can also vary with height in the biofilm. The prey cells are predominantly localized in the deeper regions of the biofilm. C) For the biofilms resulting from the competition of Δ*vasK* vs. Δ*ei* strains, heatmaps show the spatiotemporal change in parameters, analogous to panel B. Top (C1): the fraction of the biofilm biovolume occupied by the Δ*vasK* strain is shown in color. Bottom (C2): Total fraction of the image occupied by the biofilm (Δ*vasK* and Δ*ei* cells together) is shown in color. D) For the biofilms resulting from the competition of Δ*vxrB* vs. Δ*ei* strains, heatmaps show the spatiotemporal change of parameters. Top (D1): the fraction of the biofilm biovolume occupied by the Δ*vxrB* strain is shown in color. Bottom (D2): Total fraction of the image occupied by the biofilm (Δ*vxrB* and Δ*ei* cells together) is shown in color.
