# Supplementary material for: VxrB Influences Antagonism within Biofilms by Controlling Competition through Extracellular Matrix Production and Type 6 Secretion
Source: mBio. 2022 Jul 26;13(4):e01885-22. doi: 10.1128/mbio.01885-22 (PMC9426512; doi:10.1128/mbio.01885-22)
Supplement: FIG S4 [file mbio.01885-22-s0004.pdf]

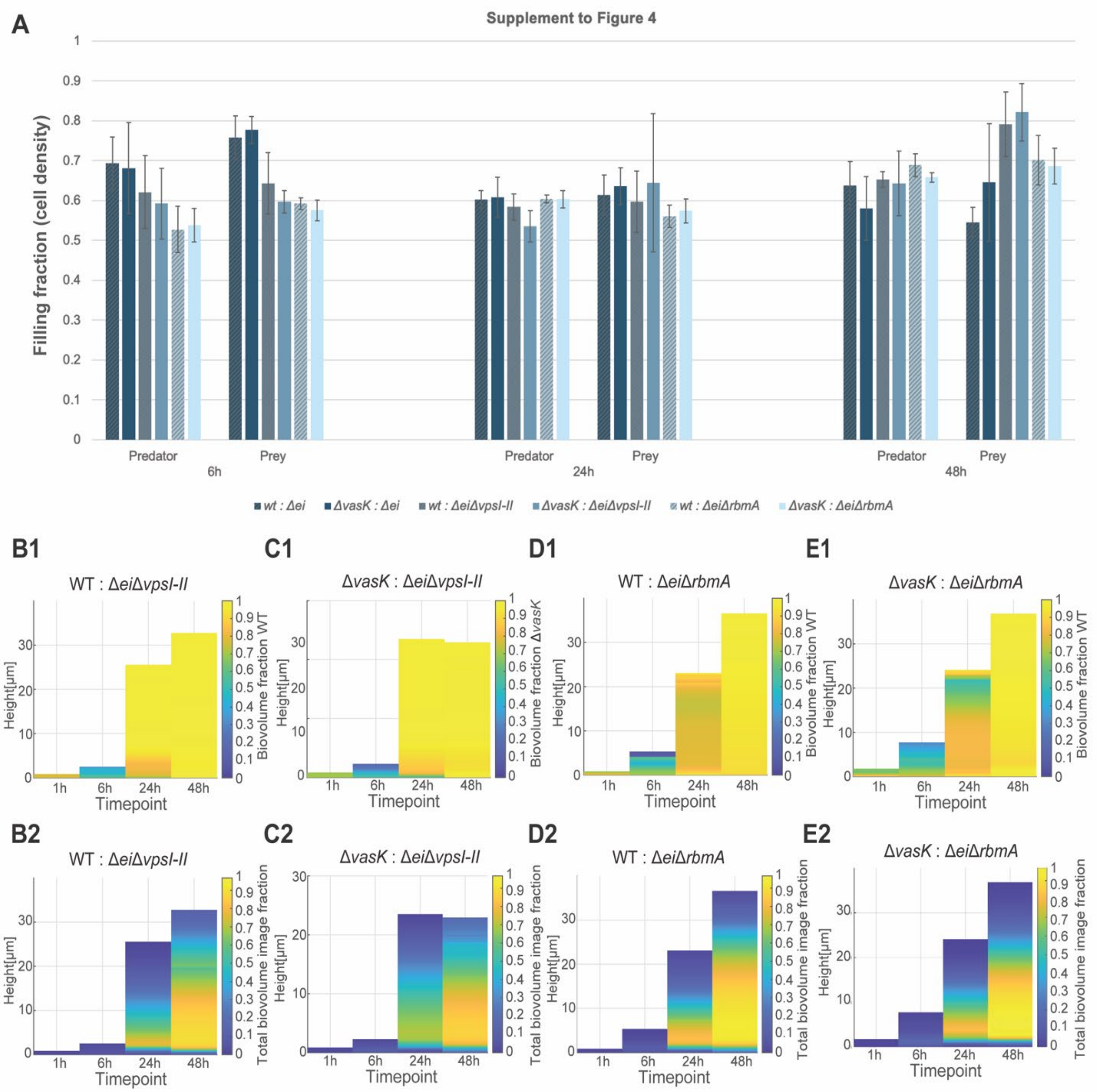

Figure S4. Quantifications of biofilm structure and strain composition for experiments from Figure 4. A) Biofilm filling fractions of regions occupied by predator cells and regions occupied by prey cells from the experiments shown in Figure 4, calculated for each strain separately. The biofilm filling fraction for each strain is the detected biovolume divided by the volume enclosed by the hull of the biovolume of each strain. No significant differences in filling fractions between the strains and time points are apparent. At the 1h time point, there were primarily individual cells, and no multicellular structures, so that the filling fraction is not well defined and therefore these data are not shown. The expected lower filling fraction for the Δ*ei*Δ*rbmA* mutant prey is not reflected in the graph as a result of the invasion of the prey-intercell-space by the predator cells. B) For the biofilms resulting from the competition of WT vs. Δ*ei*Δ*vpsI-II* strains, heatmaps show the spatial (y-axis, height inside the biofilm) and temporal (x-axis) change of parameters. Top (B1): the fraction of the biofilm biovolume occupied by the WT strain is shown in color. Bottom (B2): Total fraction of the image occupied

by the biofilm (WT and Δ*ei*Δ*vpsI-II* cells together) is shown in color, as a function of height in the biofilm. The heatmaps show that there is less biomass in higher regions of the biofilm, and that the WT fraction of the biomass can also vary with height in the biofilm. The prey cells are predominantly localized in the deeper regions of the biofilm. The Δ*ei*Δ*vpsI-II* cells are outcompeted by the WT in a stronger manner than the Δ*ei* cells in the WT vs. Δ*ei* condition (see supplementary figure S2), indicating that VPS production plays an important role in the protection against T6SS-mediated killing in biofilms. C) For the biofilms resulting from the competition of Δ*vasK* vs. Δ*ei*Δ*vpsI-II* strains, heatmaps show the spatiotemporal change in parameters, analogous to panel B. Top (C1): the fraction of the biofilm biovolume occupied by the Δ*vasK* strain is shown in color. Bottom (C2): Total fraction of the image occupied by the biofilm (Δ*vasK* and Δ*ei*Δ*vpsI-II* cells together) is shown in color. D) For the biofilms resulting from the competition of WT vs. Δ*ei*Δ*rbmA* strains, heatmaps show the spatiotemporal change in parameters, analogous to panel B. Top (D1): the fraction of the biofilm biovolume occupied by the WT strain is shown in color. Bottom (D2): Total fraction of the image occupied by the biofilm (WT and Δ*ei*Δ*rbmA* cells together) is shown in color. The Δ*ei*Δ*rbmA* cells are outcompeted by the WT in a stronger manner than the Δ*ei* cells in the WT vs. Δ*ei* condition (see supplementary figure "supplement for figure 2"), but not as strongly as in the WT vs. Δ*ei*Δ*vpsI-II* condition (panel B of the present figure). These results suggest that increased intercell-spacing due the lack of the matrix protein RbmA enhances T6SS-mediated killing in biofilms, but high cell packing is not the predominant protective mechanism as compared to VPS production. E) For the biofilms resulting from the competition of Δ*vasK* vs. Δ*ei*Δ*rbmA* strains, heatmaps show the spatiotemporal change in parameters, analogous to panel B. Top: the fraction of the biofilm biovolume occupied by the Δ*vasK* strain is shown in color. Bottom: Total fraction of the image occupied by the biofilm (Δ*vasK* and Δ*ei*Δ*rbmA* cells together) is shown in color.
