# Supplementary material for: VxrB Influences Antagonism within Biofilms by Controlling Competition through Extracellular Matrix Production and Type 6 Secretion
Source: mBio. 2022 Jul 26;13(4):e01885-22. doi: 10.1128/mbio.01885-22 (PMC9426512; doi:10.1128/mbio.01885-22)
Supplement: FIG S5 [file mbio.01885-22-s0005.pdf]

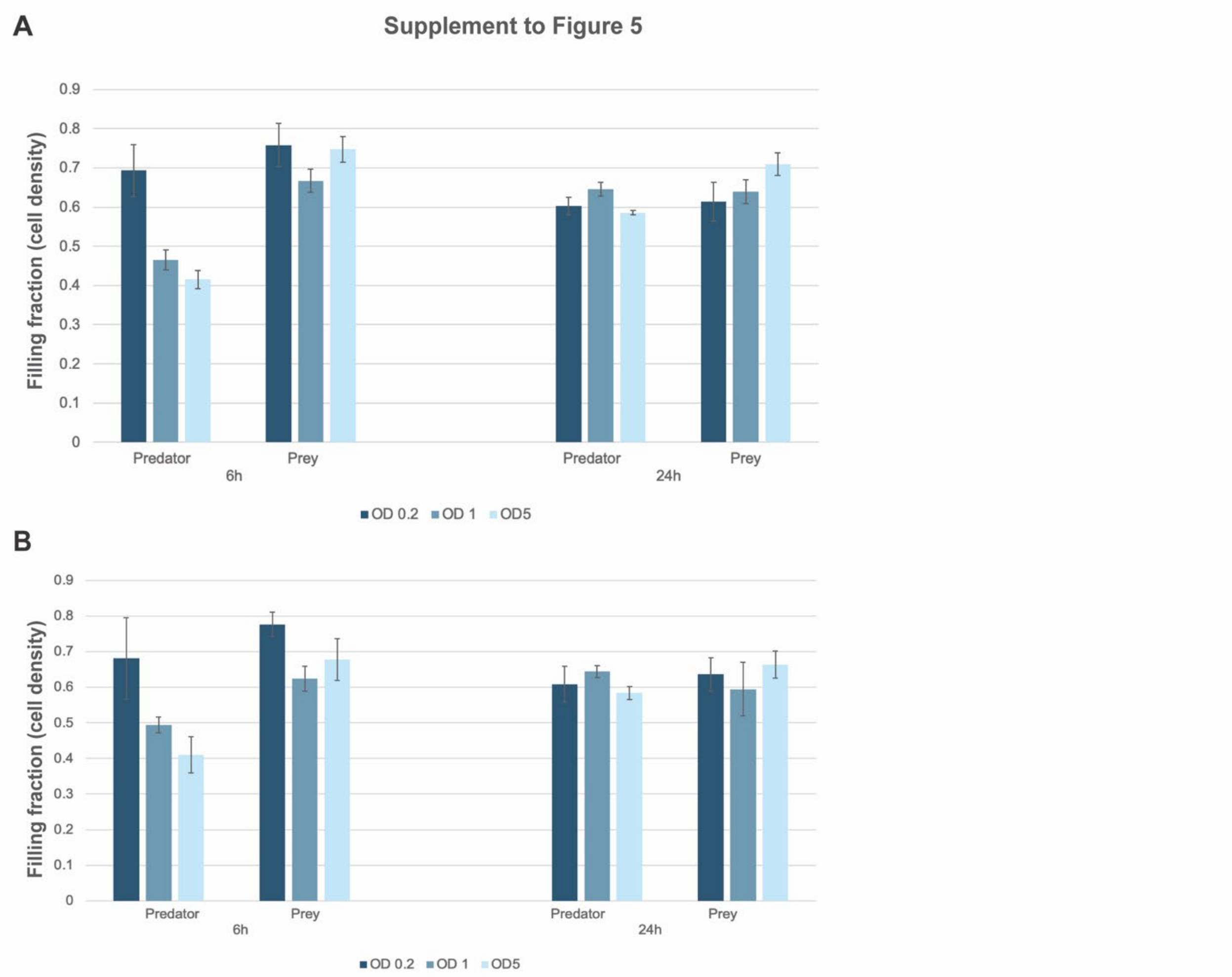

Figure S5. Quantifications of biofilm structure and strain composition for experiments from Figure 5. A) Biofilm filling fractions of regions occupied by predator cells and regions occupied by prey cells from the experiments shown in Figure 5 in the WT predator condition, calculated for each strain separately. The biofilm filling fraction for each strain is the detected biovolume divided by the volume enclosed by the hull of the biovolume of each strain. No significant differences in filling fractions between the different conditions (differing in initial seeding density of the cells in the microfluidic channel) and time points are apparent at 24h. Low filling fraction at 6h for OD 1 and OD 5 result from unclear cell cluster identification due to the high seeding density. At the 1h time point, there were primarily individual cells, and no multicellular structures, so that the filling fraction is not well defined and therefore these data are not shown.

B) Biofilm filling fractions of regions occupied by predator cells and regions occupied by prey cells from the experiments shown in Figure 5 in the ∆*vasK* predator condition, calculated for each strain separately. The biofilm filling fraction for each strain is the detected biovolume divided by the volume enclosed by the hull of the biovolume of each strain. No significant differences in filling fractions between the different conditions (differing in initial seeding density of the cells in the microfluidic channel) and time points are apparent at 24h. Low filling fraction at 6 h for OD 1 and OD 5 result from unclear cell cluster identification due to the high seeding density. At the 1h time point, there were primarily individual cells, and no multicellular structures, so that the filling fraction is not well defined and therefore these data are not shown.
