# Supplementary material for: VxrB Influences Antagonism within Biofilms by Controlling Competition through Extracellular Matrix Production and Type 6 Secretion
Source: mBio. 2022 Jul 26;13(4):e01885-22. doi: 10.1128/mbio.01885-22 (PMC9426512; doi:10.1128/mbio.01885-22)
Supplement: FIG S6 [file mbio.01885-22-s0006.pdf]

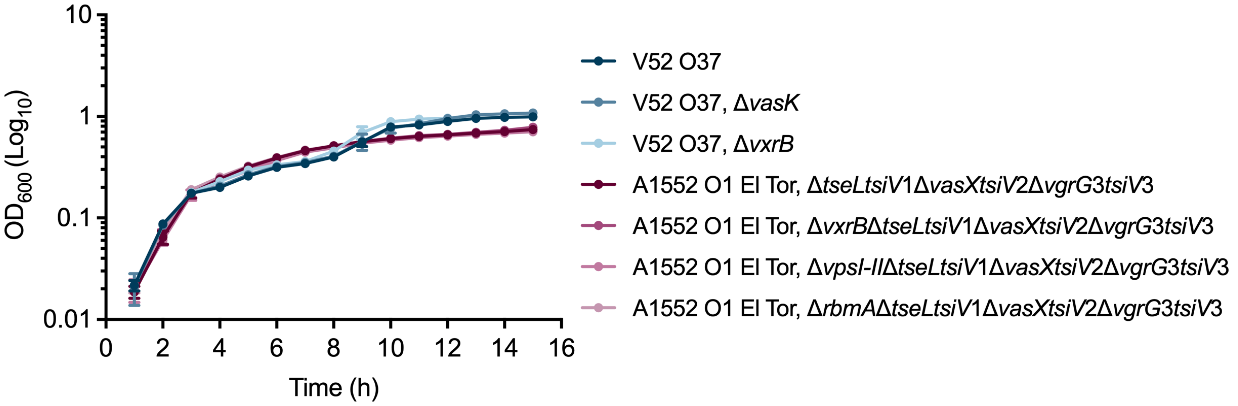

Figure S6. Growth curve of predator and prey strains. Growth curves performed in a 96 well plate over 16 hours in LB media demonstrate that there is no difference in growth rate or final culture density between predator and prey strains.
