# Supplementary material for: VxrB Influences Antagonism within Biofilms by Controlling Competition through Extracellular Matrix Production and Type 6 Secretion
Source: mBio. 2022 Jul 26;13(4):e01885-22. doi: 10.1128/mbio.01885-22 (PMC9426512; doi:10.1128/mbio.01885-22)
Supplement: FIG S7 [file mbio.01885-22-s0007.pdf]

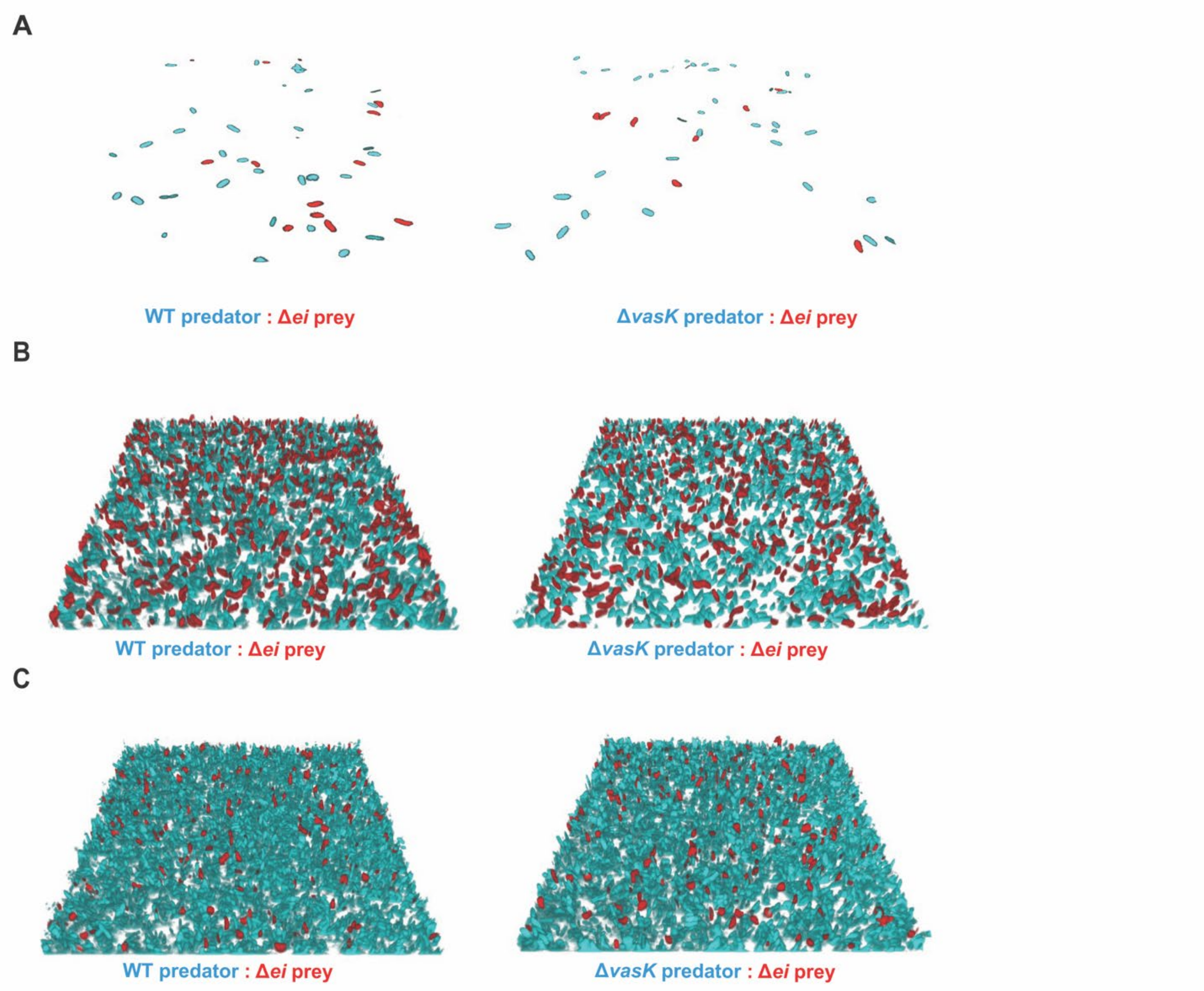

Figure S7. Predator and prey strain initial attachment after 1 hour incubation using different inoculation ODs. A) Predator (blue) and prey (red) attachment to surface after 1 hour when inoculating with an initial inoculum OD_{600}=0.02. B) Predator (blue) and prey (red) attachment to surface after 1 hour when inoculating with an initial inoculum OD_{600}=1. C) Predator (blue) and prey (red) attachment to surface after 1 hour when inoculating with an initial inoculum OD_{600}=5.
